# The HOPE fixation technique - a promising alternative to common prostate cancer biobanking approaches

**DOI:** 10.1186/1471-2407-11-511

**Published:** 2011-12-07

**Authors:** Martin Braun, Roopika Menon, Pavel Nikolov, Robert Kirsten, Karen Petersen, David Schilling, Christina Schott, Sibylle Gündisch, Falko Fend, Karl-Friedrich Becker, Sven Perner

**Affiliations:** 1 Institute of Pathology, Comprehensive Cancer Center, University Hospital of Tuebingen, Tuebingen, Germany; 2 Department of Urology, Comprehensive Cancer Center, University Hospital of Tuebingen, Tuebingen, Germany; 3 Institute of Pathology, Technical University of Munich, Munich, Germany; 4 Institute of Prostate Cancer Research, Institute of Pathology, University Hospital Bonn, Sigmund-Freud-Straße 25, 53127 Bonn, Germany

**Keywords:** HOPE technique, HOPE fixation, Prostate cancer

## Abstract

**Background:**

The availability of well-annotated prostate tissue samples through biobanks is key for research. Whereas fresh-frozen tissue is well suited for a broad spectrum of molecular analyses, its storage and handling is complex and cost-intensive. Formalin-fixed paraffin-embedded specimens (FFPE) are easy to handle and economic to store, but their applicability for molecular methods is restricted. The recently introduced Hepes-glutamic acid-buffer mediated Organic solvent Protection Effect (HOPE) is a promising alternative, which might have the potential to unite the benefits of FFPE and fresh-frozen specimen. Aim of the study was to compare HOPE-fixed, FFPE and fresh-frozen bio-specimens for their accessibility for diagnostic and research purposes.

**Methods:**

10 prostate cancer samples were each preserved with HOPE, formalin, and liquid nitrogen and studied with in-situ and molecular methods. Samples were H&E stained, and assessed by immunohistochemistry (i.e. PSA, GOLPH2, p63) and FISH (i.e. *ERG *rearrangement). We assessed DNA integrity by PCR, using control genes ranging from 100 to 600 bp amplicon size. RNA integrity was assessed through qRT-PCR on three housekeeping genes (TBP, GAPDH, β-actin). Protein expression was analysed by performing western blot analysis using GOLPH2 and PSA antibodies.

**Results:**

Of the HOPE samples, morphologic quality of H&E sections, immunohistochemical staining, and the FISH assay was at least equal to FFPE tissue, and significantly better than the fresh-frozen specimens. DNA, RNA, and protein analysis of HOPE samples provided similar results as compared to fresh-frozen specimens. As expected, FFPE-samples were inferior for most of the molecular analyses.

**Conclusions:**

This is the first study, comparatively assessing the suitability of these fixation methods for diagnostic and research utilization. Overall, HOPE-fixed bio-specimens combine the benefits of FFPE- and fresh-frozen samples. Results of this study have the potential to expand on contemporary prostate tissue biobanking approaches and can serve as a model for other organs and tumors.

## Background

The importance of prostate tissue bio-repositories is increasing as they are forming an invaluable resource of samples for profound translational research [1-4]. Long term and, preferably, native preservation is an essential requirement of stored samples. Due to the nature of prostate carcinomas which commonly develops multiple and independent tumor foci, they are difficult to identify macroscopically. For research purposes, it is recommended to fresh-freeze each second prostate slice in order to capture the tumour with all foci [2,5-7]. Thus, comprehensive prostate cancer biobanking requires significant amounts of resources, and large patient counts would eventually exhaust the bio-repository in a short period of time. In conflict with the recommendation of prostate tissue biobanking, current diagnostic guidelines demand the embedding of the complete prostate for routine diagnosis, almost exclusively conducted via formalin-fixation and subsequent paraffin-embedding (FFPE). This leads to a restriction in the amount of available quality tissue for research.

Whilst the limitedly available fresh-frozen tissue is most applicable to a broad spectrum of molecular analyses, its storage and handling is complex and cost-intensive. On the other hand, the abundantly available FFPE specimens are easy to handle and economic to store, but their applicability for modern analysis methods is restricted [7-12]. The recently introduced, formalin-free Hepes-glutamic acid buffer mediated Organic solvent Protection Effect (HOPE) fixation method is a promising alternative, which might have the potential to unite the benefits of FFPE and fresh-frozen specimen. By this means, the HOPE fixed specimen may have the potential to overcome the problems being faced by both the research and diagnostics. HOPE-fixed specimens have been used for several studies and their viability has been assessed on different human tissues using several molecular methods [13-24]. However, no studies have been reported so far, parallely assessing the advantages and disadvantages of FFPE, fresh-frozen and HOPE-fixed prostate specimens. Thus, the aim of our study was to comprehensively compare fresh-frozen samples, HOPE-fixed samples, and FFPE samples for their application to common morphologic and molecular methods (i.e. H&E staining, immuhistochemistry, DNA and RNA extraction, PCR, qRT-PCR, western blotting and fluorescence in-situ hybridization). Results of this study could have the potential to expand on contemporary prostate tissue biobanking approaches.

## Methods

### Material

All experiments were performed on a radical prostatectomy cohort of 10 patients diagnosed and treated at the University Hospital of Tubingen, Germany. For each patient, we identified normal prostatic tissue and prostate cancer tissue from the 10 corresponding prostatectomy samples.

Benign and cancerous prostatic tissues were separated into three portions of equal size. Subsequently, the three portions were fixed using the conventional FFPE protocol, the recently introduced HOPE-fixation, and cryo-conservation method. HOPE-fixation was been performed as described earlier [18]. For detailed protocols, refer to supplementary information (see Additional file 1).

### H&E staining and immunohistochemistry

For H&E staining and immunohistochemistry, 2.5 μm sections of all fixed samples were mounted on superfrost slides. For immunohistochemistry pre-treatment, the FFPE and HOPE sections were deparaffinised, using EZ Preparation Buffer, pH 7.0, followed by stabilization reaction with TRIS Buffer, pH = 7.6-7.8 (Ventana Medical Systems, Tucson, AZ, USA). Immunohistochemistry was conducted with the Ventana Benchmark automated staining system (Ventana Medical Systems, Tucson, AZ, USA) using Ventana reagents. For immunohistochemical staining, the following clones and primary antibodies were used: prostate-specific antigen (PSA) antibody (clone 35H9, Novocastra, Newcastle, UK, dilution 1:50), GOLPH2 antibody (clone 5B10; Abnova, Heidelberg, Germany, dilution 1:1000), p63 antibody (clone 4A4; DAKO, Haverlee, Belgium, dilution 1:100). No epitope retrieval has been performed for the HOPE and fresh-frozen sections. Multiple board-certified pathologists did the evaluation of the slides independently. Criteria for a sufficient staining were antibody binding specificity, tissue morphology and overall staining quality. For a detailed break-up, refer to the supplement (see Additional file 1).

### Fluorescence in situ hybridization

We used fluorescence in-situ hybridization (FISH) assay to detect the *ERG *rearrangement at the chromosomal level on each specimen [25,26]. Hence, we performed a split-signal-approach, with two probes spanning the *ERG *locus as described earlier. For FISH assays, 5 μm sections of all samples were mounted on superfrost slides. All cases were independently assessed by two experienced evaluators (M.B., and S.P.) At least 100 nuclei per case were evaluated. Criteria for an efficient FISH staining were clear distinguishable cell morphology with strong distinct *ERG *signals within the nuclei, and minimal background signals. See full protocol in the supplement (see Additional file 1).

### DNA extraction

DNA extraction was performed using the routine Phenol:Choroloform: Isoamylalcohol (PCI) method. For subsequent DNA quality assessment using PCR, we chose 6 FFPE specimens, along with their corresponding HOPE and fresh-frozen samples, each having a sufficient amount of DNA and a high integrity value for all the three preservation methods. See full protocol in the supplement (see Additional file 1).

### PCR

For the assessment of amplifiability and integrity of DNA samples a control gene PCR was performed using five control genes exon and five primer sets for obtaining PCR products ranging from 100 to 600 bp. This was followed by a gel electrophoresis to analyse the bands of the corresponding PCR products. As reported by Van Dongen et al. the presence of five distinct bands for the control genes, ranging from 100 to 600 bp, is a good representation of DNA integrity and amplifiability. For a detailed break-up, refer to supplement (see Additional file 1).

### RNA extraction

Tissue sections were prepared, using a RNAse-free microtome and 1.5 ml RNAse-free tubes. For FFPE and HOPE fixed tissues, xylene and ethanol deparaffinization step was performed according to the manufacturer's recommendations. For each block, the topmost five sections were discarded and only the adjoining deeper sections (ten per sample) were then cut and deparaffinised. RNA was isolated using the column-based purification protocol with QIAGEN RNeasy FFPE kit (QIAGEN GmbH, Hilden, Germany).

RNA integrity and quantity was estimated using the NanoDrop 1000 C Spectrophotometer (NanoDrop Technologies, Wilmington, DE, USA). Residual DNA was destroyed by treatment with DNase (Qiagen). For quantitative RT-PCR, we chose the six most promising FFPE and corresponding HOPE and fresh-frozen specimens, having a sufficient amount of RNA and a high integrity value.

### Quantitative RT-PCR

Quantitative RT-PCR analysis was performed with the Roche Light Cycler 480 (Roche, Switzerland). cDNA was synthesized by reverse transcriptase (Revert Aid H Mini First Strand cDNA Synthesis Kit, Fermentas Life Sciences) using 100 ng of total RNA and an oligo dT15 primer provided in the kit. We quantified three house-keeping genes (TBP, GAPDH, β-actin) with varying amplicon lengths to check for RNA integrity from FFPE, HOPE and fresh frozen samples. See full protocol in supplement (see Additional file 1).

### Protein extraction and western blot

Protein extraction was performed on FFPE, HOPE and fresh-frozen samples [27,28] and protein concentrations were determined using the Bradford protein assay (BioRad Protein Assay (# 500-0006)). Equal amounts of protein lysates (18 μg) were separated by one-dimensional SDS-polyacrylamide (10%) gel electrophoresis and blotted onto nitrocellulose membranes (Schleicher and Schuell, Dassel, Germany). Protein detection was performed using anti-β-Actin (Sigma A1978 AC15, 1:10.000 in 5% milkpowder/TBST), anti-PSA (Novocastra #NCL-PSA-431, 1:1000 in 5% milkpowder/TBST) and anti-GOLPH2 antibodies (Abnova #H00051280-M06, 1:1000 in 5% milkpowder/TBST). See a detailed protocol in the supplement. Western blot quantification was performed using Scion Image software (Scion Corp., Frederick, MD, USA). For protein yield calculation the area of the sections was measured using AxioVision Rel. 4.8 (Carl Zeiss Imaging Solutions GmbH, Göttingen, Germany), and the yield (X) was calculated as follows:

$$\text{Total}\;\text{protein}\;\text{amount}∕\left(\text{area}\;*\;\text{section}\;\text{thickness}*\text{number}\;\text{of}\;\text{sections}\right)=X\;\mu \text{g}∕\text{m}{\text{m}}^{3}$$

### Statistical analysis

For statistical analysis SPSS Statistics 19 was used (SPSS Inc., Chicago, IL). In order to assess the differences in performance in the three fixation subsets, we performed the Student's *t*-test and Friedman test. A p-value lower than 0.05 was considered to be statistically significant.

## Results

Prior to DNA and RNA extraction and evaluation, specimens were examined on a histological basis. Conventionally used pathological techniques, such as HE staining, immunohistochemistry (IHC) and FISH were carried out for all samples (i.e. FFPE-fixed specimens, HOPE-fixed specimens, fresh-frozen specimens). HOPE specimens constantly displayed a 'FFPE-like' histomorphology with specific signals for each of the above mentioned techniques. As expected, fresh frozen specimens showed typical freeze-related artefacts regarding histologic quality, such as freeze artefacts, partially distorted morphology, and interfering background.

**Figure 1:**
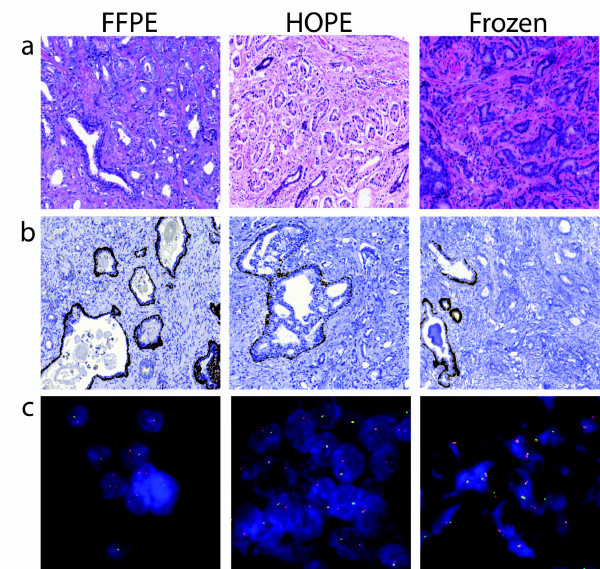
**Morphological quality comparison between FFPE, HOPE, and fresh-frozen specimens**. **a**. Representative HE stained sections of FFPE, HOPE, and fresh frozen material. Tissue sections of HOPE fixed prostate cancer specimens show a 'FFPE -like' morphology. As expected, frozen tissue displayed typical indistinct cytomorphology. **b**. Representative p63 antibody immunohistochemistry staining of FFPE, HOPE, and fresh frozen material. FFPE and frozen sample display benign glands and infiltrating prostate cancer glands. The HOPE sample depicts infiltrating tumor glands and an intraductal spread of the prostate cancer. In the FFPE and frozen samples, the p63 antibody highlights the basal cells of the normal prostatic glands. In the HOPE sample, the p63 antibody highlights the basal cells of the gland with intraductal tumor spread. In all samples, the infiltrative prostate cancer glands are negative for p63 staining. The efficient binding specificity and signal strength of all samples is comparable, while the histomorphologic quality is inferior in the frozen sample. **c**. Representative Fluorescence in-situ hybridisation of *ERG *rearrangement on FFPE, fresh frozen and HOPE tissue sections. High power magnification. FISH for *ERG *on formalin-fixed, paraffin-embedded prostate cancer tissue. As expected for fresh-frozen samples, signals are specific, but the tissue section exhibits a typical freeze-related clumping of cells with a lack of distinct morphology. On the other hand, FISH for *ERG *on HOPE fixed prostate cancer tissue show a 'FFPE-like' signal specificity and morphology.

IHC staining of HOPE samples was time-efficient compared to the FFPE specimens, as no epitope retrieval was necessary. However, unlike the HOPE-manufacturer's recommendation for the FISH assay, a brief proteinase pretreatment was needed for HOPE specimens to enhance signal quality. The results are displayed and summarized in Figure 1

**Figure 2:**
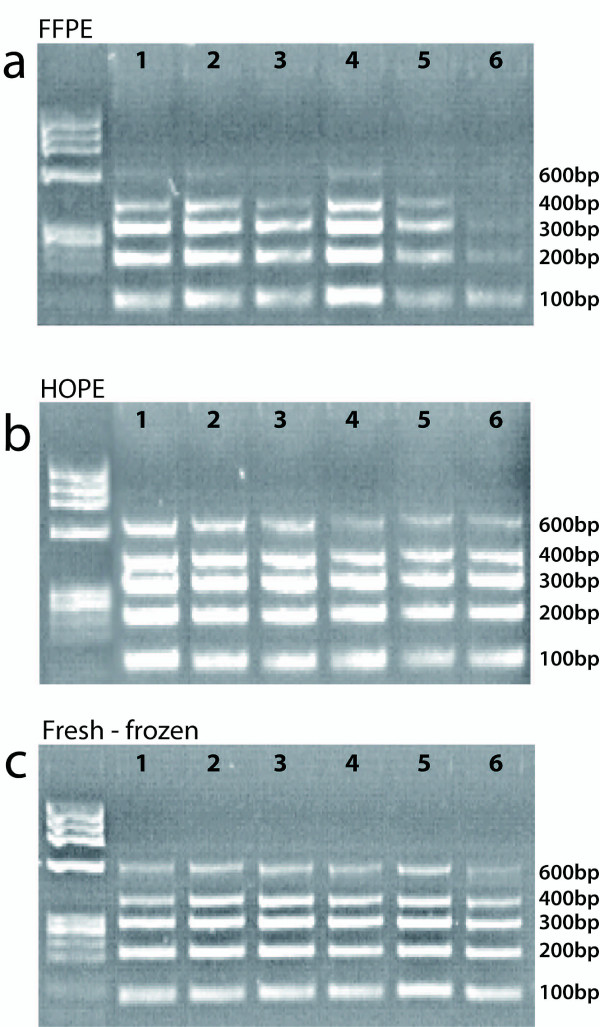
**DNA integrity of FFPE, HOPE, and fresh-frozen tissues**. Control gene PCR for the assessment of amplification and integrity of DNA samples obtained from FFPE, HOPE, and fresh frozen tissues. Gel electrophoresis of five control gene exon PCR products ranging from 100 to 600 bp. **a**. FFPE derived DNA samples. Clear bands were observed for 4 control genes. The 600 bp band was visible only in lane 6, suggesting a certain amount of DNA degradation. **b**. HOPE derived DNA samples. Clear bands were observed for all 5 control genes. Results were comparable to the DNA extracted from fresh frozen samples, suggesting good DNA integrity. **c**. Fresh frozen derived DNA samples. Clear bands were observed for all 5 control genes suggesting good DNA integrity, as expected.

To analyze the feasibility to amplify the extracted DNA, we performed a PCR with a set of five primers of different sizes and analyzed all the amplicons on a 2% agarose gel, stained with ethidium bromide. Depending on the fixation method, we observed differences in the DNA quality. A total of the mentioned five bands were expected - 100 bp, 200 bp, 300 bp, 400 bp, and 600 bp. All samples effectively amplified bands of up to 400 bp. HOPE and fresh-frozen specimens displayed all five distinct bands, while the FFPE samples showed the absence of the 600 bp band, as depicted in Figure 2a-c.

To compare the potential of the different fixation techniques for the detection and quantification of RNA, real time RT-PCR was performed. RNA was extracted from six patient samples, i.e. six HOPE specimens and their corresponding FFPE and fresh-frozen specimens. The reverse transcribed cDNA, obtained from the extracted RNA, was then used to detect the C_t _value. A quantitative RT-PCR of three housekeeping genes (TBP, GAPDH and β-actin) of different amplicon sizes was performed for all specimens.

**Figure 3:**
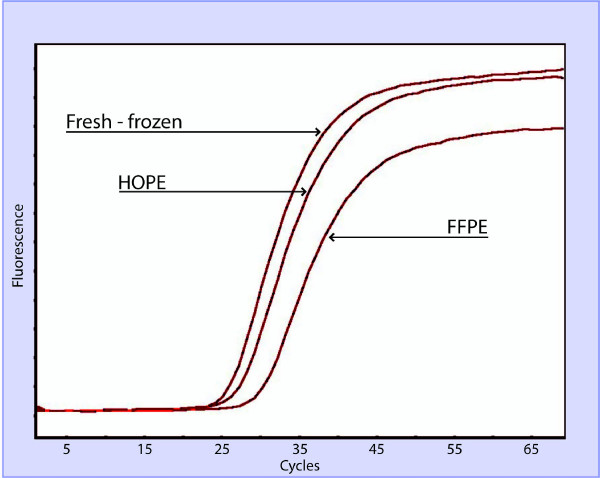
**RNA integrity of FFPE, HOPE, and fresh-frozen tissues**. Representative real-time RT-PCR targeting *TATA*-binding-protein (TBP, 73 bp) in FFPE, HOPE, and fresh-frozen tissues. The threshold value of HOPE fixed specimens was comparable to that of the fresh-frozen specimen.

As expected, on an average, the cycle threshold (C_t_) derived from the fresh frozen specimens, naturally lacking any fixative interference, showed the lowest C_t _values, followed by HOPE and FFPE specimens. (Figure 3) The average C_t _threshold HOPE values for TBP, GAPDH and β-actin were 29.2, 27.8, and 27.6 respectively. The average Ct threshold FFPE values for TBP, GAPDH and β-actin were 33.2, 34.5, and 35.5, respectively. The average C_t _threshold of fresh-frozen specimens values for TBP, GAPDH and β-actin were 27.1, 26.4, 25.8 respectively. Of note, these differences in the average C_t _were statistically significant (*p *< 0.05, *t*-test). See Table 1 for a detailed break-up of the different C_t _values.

**Table 1:** RNA Quality Assessment.

TBP		
**FFPE**	**HOPE**	**Native**

29,9	27,15	25,5

31,89	29,18	27,2

34,8	29,8	28,1

33,6	29,7	27,5

33,1	29,9	26,3

35,86	29,6	28,2

GAPDH		

FFPE	HOPE	Native

29,4	25,5	25,1

35,8	29,7	27,4

37,7	27,9	25,6

31,7	28	27,3

36,2	27,9	25,7

35,9	27,8	27,1

		

Beta actin		

FFPE	HOPE	Native

36,9	28,2	26,7

38,7	25,6	24,1

31,8	31,1	25,8

34,2	26	25,5

33,2	29,3	28,1

38,12	25,7	24,8

Cycle threshold values for TBP, GAPDH and ß-actin from the corresponding FFPE, HOPE, and fresh-frozen samples.

**Figure 4:**
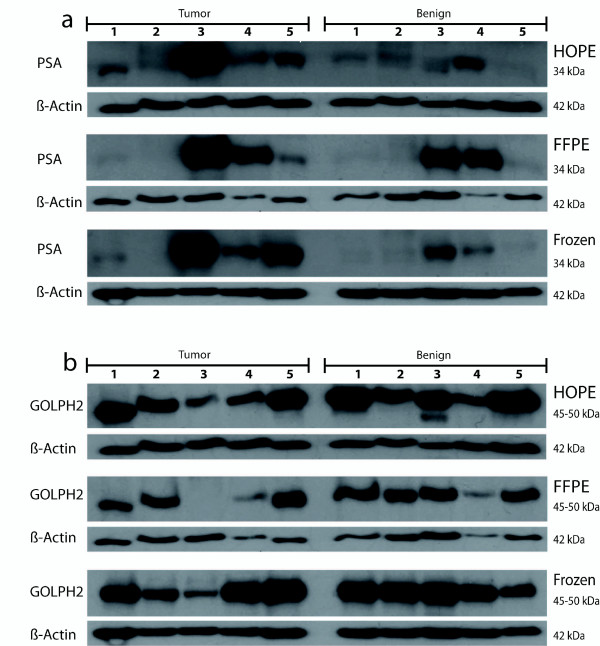
**Western blot analysis of FFPE, HOPE, and fresh-frozen samples using PSA (34 kDa), GOLPH2 (45-50 kDa), and beta-actin (42 kDa) antibodies**. Eighteen μg of protein was separated by SDS-PAGE and western blot analysis was performed using indicated antibodies. Strong bands could be detected at 34 kDa with the anti-PSA antibody (**a**) and at 45-50 kDa with the anti-GOLPH2 antibody (**b**). The housekeeping gene beta-actin also showed clear bands at 42 kDa. Signal intensities of HOPE-fixed tissue specimens were at least as intense as those of cryopreserved and FFPE samples.

**Figure 5:**
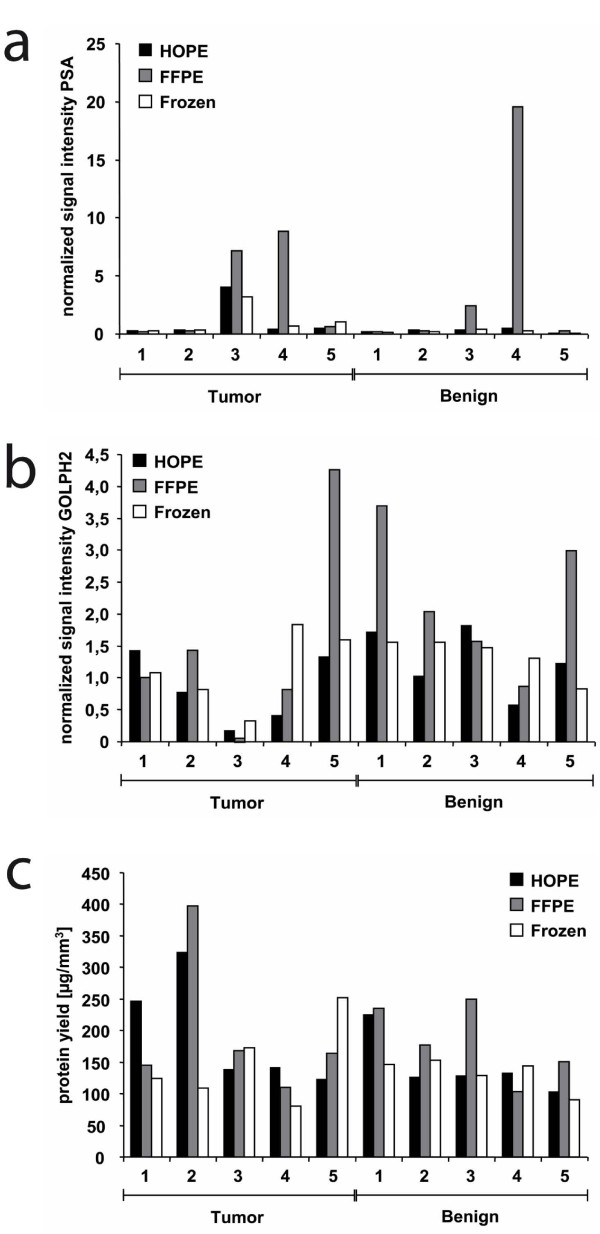
**Western blot quantification and protein yield calculations**. Western blot quantification was performed using Scion Image software. Depicted are the signal intensities of anti-PSA (**a**) and anti-GOLPH2 (**b**) antibodies, normalized to beta-Actin. The quantification of the western blots revealed for most of the samples no differences between the signal intensities from cryopreserved, HOPE-fixed or formalin-fixed samples. FFPE showed for some cases even the strongest signal which might be dependent on the epitope detected by the specific antibody or due to a very weak beta-Actin staining for those FFPE samples. For protein yield calculations (**c**) the area of the sections was measured using AxioVision Rel. 4.8 and the yield was calculated for each sample. The resulting protein yields were similar or even higher for HOPE and FFPE tissues compared to cryopreserved samples.

To compare protein quality and quantity, western blot analysis and protein yield calculations were performed. In the western blot analysis we detected strong signals at 34 kDa, 45-50 kDa and 42 kDa using antibodies against PSA, GOLPH2 and beta-Actin, respectively, independent of the preservation method. We obtained clear bands for HOPE-fixed tissue specimens which were at least as intense as those of cryopreserved and FFPE samples. Interestingly, in the tumor sample of case #3 high PSA and low GOLPH2 expression could be detected, independent of the preservation method (Figure 4). The quantification of the western blots and normalization against beta-Actin revealed for most of the samples no significant differences between the signal intensities from cryopreserved, HOPE-fixed or formalin-fixed samples. FFPE showed for some cases even the strongest signal which might be dependent on the epitope detected by the specific antibody or due to a very weak beta-Actin staining for those FFPE samples (Figure 5a and 5b). The resulting protein yields were similar or even higher for HOPE and FFPE tissues compared to cryopreserved samples. However, by taking into account all cryopreserved, FFPE, and HOPE samples no statistically significant differences could be observed (*p *= 0.3, Friedman test) (Figure 5c).

## Discussion

The availability of well-annotated human prostatic tissue samples through biobanks is key for translational research [1-4]. Due to the nature of prostate carcinomas developing multiple and independent tumor foci, which are tedious to identify macroscopically, it is recommended to freeze each second prostate tissue slice. Thus, comprehensive prostate cancer biobanking requires enormous amounts of resources [2,5-7]. However, this is in conflict with the existing guidelines requiring all prostatic tissue to be used for diagnostic purposes. By this means, the amount of fresh-frozen tissue available for research is limited. FFPE tissues, as used in diagnostics, are easy to handle and economic to store, but their applicability for subsequent research is restricted [7-12]. On the other hand, the sparsely available fresh-frozen tissue is most applicable to a broad spectrum of molecular analyses, but its storage and handling is complex and cost-intensive. The recently introduced, formalin-free HOPE-fixation method is a promising alternative, having the potential to unite the benefits of FFPE and fresh-frozen specimens. This fixation method may be able to bridge the above-described gap of available, superior quality tissue, and thus resolve the conflict faced by both diagnostic and research. Recently, HOPE-fixed material has been used in few studies and the feasibility of this fixation technique has been assessed on different human tissues [13-24].

This is the first comparative study simultaneously analysing the molecular and morphological integrity of FFPE, HOPE and fresh-frozen prostate cancer specimens from the same samples. Our results for the HOPE fixed specimens suggested that problematic factors such as distinct nucleic acid degradation and methylene cross linking are absent. In terms of morphology, the HOPE fixed specimens displayed a superior and formalin-like quality. In addition, for IHC assessment of HOPE samples, no antigen-retrieval was required. For in-situ analysis, even though the manufacturer recommended no enzymatic digestion, our study showed that the FISH signals in HOPE samples benefited with enzymatic pretreatment. Furthermore, DNA, RNA and protein integrity of the HOPE samples were comparable to the fresh-frozen samples, yielding far better results than the FFPE specimens for most of the approaches investigated in this study. PCR results indicate that DNA extracted from HOPE, FFPE and fresh-frozen samples is suitable for downstream molecular applications. As expected, DNA isolated from FFPE specimens was highly fragmented as compared to the HOPE and fresh-frozen specimens, but could be used for successful amplification of shorter amplification products up to 400 bp in length. Thus, on the molecular basis, HOPE fixation has proven to be promising, providing similar results as fresh-frozen specimens, without significant reduction in quality or the amount of extractable nucleic acids. On the same hand, HOPE fixed specimens equally displayed 'FFPE-like' morphology. However, HOPE fixation has certain limitations. Manufacturer recommends storage of HOPE fixed specimens at 4°C. Extensive studies have not been performed on long-term storage issues. Moreover HOPE protocols for fixation are relatively long, and tissue blocks are tougher to handle due to a lower melting point of the paraffin material. Lastly, and most importantly, the HOPE technique does not completely fix the tissue, resulting in a potential hazard of viral, prion, micro-organism infection.

Nevertheless, with regard to today's diagnostic and research pathology, HOPE fixation has a promising potential to add to the current fixation approaches. This method could easily be implemented in the research and diagnostic surroundings, as the same instruments are needed as required for FFPE processing and sectioning. On the other hand, regarding costs, FFPE is by far the cheapest method to preserve tissue specimens. However, HOPE fixation is cheaper than cryopreservation, especially when it comes to numerous samples. This is due to the fact that HOPE samples can economically be stored in refrigerators, are easier to handle, and do not have space constraints due to the cassette frames. Nonetheless, a remaining limiting factor might be the amount of time needed for the manual HOPE fixation protocol as compared to the widely used and highly standardized automatic FFPE protocol. But to our knowledge, an automated HOPE fixation device is currently available in the market.

Lastly, the fixation protocols followed in our bio-repository may not necessarily be identical to fixation approaches applied elsewhere. Consequently, follow up studies using a larger cohort are needed to address these aspects in detail, such as the impact of different conditions (e.g. time, concentration, temperature, etc.), on quality and integrity of the specimens.

## Conclusions

In conclusion, this is the first study comparing in parallel HOPE fixed bio-specimens with FFPE and fresh-frozen samples for their suitability for diagnostic and research utilization. We were able to illustrate that HOPE specimens largely combine the advantages of FFPE and fresh-frozen material. Our studies show that the HOPE fixation could be a promising alternative for initial prostate tissue storage, enabling comprehensive research upon routine diagnosis. Results of this study have the potential to expand on contemporary prostate tissue biobanking approaches and can furthermore be a model for other organs and tumors.

## Competing interests

The Brigham and Women's Hospital and the University of Michigan have filed a patent on ETS gene rearrangements in prostate cancer, on which S.P. is a co-inventor and the diagnostic field of use has been licensed to GenProbe Inc. GenProbe has not played a role in the design and conduct of the study, nor in the collection, analysis or interpretation of the data and no involvement in the preparation, review or approval of the manuscript.

## Authors' contributions

SP was responsible for conception and design of the study. Experimental data was acquired by MB, RM, KB and PN. Manuscript was drafted by MB, RM, and SP. FF, DS, SG and KB performed critical revision of the manuscript and provided important intellectual content. Statistical analysis was done by MB, KB, and RM. Technical and material support was provided by KP, RM, PN, KB, CS, SG, FF, and DS. All authors read and approved the final manuscript.

## Pre-publication history

The pre-publication history for this paper can be accessed here:

http://www.biomedcentral.com/1471-2407/11/511/prepub

## Supplementary Material

Additional file 1 **Supplementary information**. Supplementary Methods and materials. Click here for file
