# Supplementary material for: The HOPE fixation technique - a promising alternative to common prostate cancer biobanking approaches
Source: BMC Cancer. 2011 Dec 7;11:511. doi: 10.1186/1471-2407-11-511 (PMC3248383; doi:10.1186/1471-2407-11-511)
Supplement: Additional file 1 — Supplementary information. Supplementary Methods and materials. [file 1471-2407-11-511-S1.DOC]

**Supplementary Information**

**Material & Methods**

Sample preservation

FFPE samples were fixed in 10% neutral buffered formalin and embedded in paraffin. HOPE-fixation was been performed as described earlier. Briefly, tissue was incubated in HOPE® I solution at 0-4°C for 12-72 hours. Subsequently, after removal of HOPE® I solution, ice-cold HOPE® II and acetone solution is added to the tissue, and incubated for 2 hours. Next, HOPE® II and acetone was removed, and pure ice-cold acetone was applied for 2 hours. This step repeated twice. Finally, low-melting paraffin was poured on the fixed tissue, and placed into a regular block cassette. HOPE-samples were then stored in a refrigerator at 4°C. Native samples were frozen using liquid nitrogen and stored at -80°C.

H&E staining

For H&E staining, 2.5 µm sections of all fixed samples were mounted on superfrost slides. H&E staining on the FFPE and HOPE specimen was performed using standard FFPE staining methodology. Fresh frozen specimens were stained following the routine staining protocol. The evaluation of the H&E slides was done independently by multiple board-certified pathologists. Criteria for a sufficient H&E staining were distinct tissue morphology with minimum artefacts.

Immunohistochemistry

For immunohistochemistry, 2.5 µm sections in thickness of all fixed samples were cut by rotation microtome and then mounted on superfrost slides. For pre-treatment, the FFPE and HOPE sections were deparaffinised, using EZ Preparation Buffer, pH 7.0, followed by stabilization reaction with TRIS Buffer, pH= 7.6- 7.8 (Ventana Medical Systems, Tucson, AZ, USA). Immunohistochemistry was conducted with the Ventana Benchmark automated staining system (Ventana Medical Systems, Tucson, AZ, USA) using Ventana reagents. For immunohistochemical staining, the following clones and primary antibodies were used: prostate-specific antigen (PSA) antibody (clone 35H9, Novocastra, Newcastle, UK, dilution 1:50), GOLPH2 antibody (clone 5B10; Abnova, Heidelberg, Germany, dilution 1:1000), p63 antibody (clone 4A4; DAKO, Haverlee, Belgium, dilution 1:100). Dilution was performed using a Ventana diluent. For all FFPE samples, the pre-treatment included heat induced epitope retrieval for immunohistochemical stainings was performed. No epitope retrieval has been performed for the HOPE and fresh-frozen sections. All slides were washed in phosphate-buffered saline and H_2_O. Slides were counterstained with haematoxylin (Ventana Medical Systems, Tucson, AZ, USA) and the proper alkalinity was ensured by washing with Bluing reagent, pH= 8.0. For FFPE and HOPE samples, additional dehydration step included initial ethanol wash cycle (70%, 80%, 96% and 100% Ethanol), followed by 4 xylene wash steps. The secondary washing was performed using CC1 buffer (Cell conditions 1) and TBE buffer with pH= 8.4 (Ventana Medical Systems, Tucson, AZ, USA). Primary antibody detection was performed using the iView™ DAB detection kit (Ventana Medical Systems, Tucson, AZ, USA). Multiple board-certified pathologists did the evaluation of the slides independently. Criteria for a sufficient staining were antibody binding specificity, tissue morphology and overall staining quality.

ERG break-apart fluorescence in situ hybridization assay

We used fluorescence in-situ hybridization (FISH) assay to detect the *ERG* rearrangement at the chromosomal level on each specimen [25-27]. Hence, we performed a split-signal-approach, with two probes spanning the *ERG* locus as described earlier. For FISH assays, 5 µm sections of all fixed samples were mounted on superfrost slides.

FISH protocol for FFPE specimen

Deparaffinized FFPE sections were pre-treated with a 100 mM Tris and 50 mM EDTA solution at 92.8C° for 15 min. and digested with Digest-All III (dilution 1:2) at 37°C for 22 min. *ERG* FISH probes were denatured at 73°C for 5 min. and immediately placed on ice. Subsequently, the tissue sections and *ERG* FISH probes were co-denatured at 94°C for 3 min. and hybridised overnight at 37°C. We used BAC clones RP11-24A11 for centromeric labeling with biotin and RP11-372O17 for telomeric labelling with digoxigenin. Posthybridisation washing was done with 2x SSC at 75°C for 7 min., and the fluorescence detection was carried out using streptavidin-Alexa-594 conjugates (dilution 1:200) and anti-digoxigenin-FITC (dilution 1:200). Slides were then counterstained with 4’,6-Diamidin-2’ phenylindoldihydrochlorid (DAPI) and mounted.

FISH protocol for HOPE specimen

To validate the manufacturer’s recommendation of the absence of enzymatic digestion, the HOPE specimens were divided into a digested and a non-digested fraction. HOPE fixed sections, mounted on superfrost slides were subjected to an initial aging step for 1 hour at 60C°, followed by another aging step for 10 min. at 70C°. The samples were subsequently washed in Xylene and Ethanol, and consequently washed in Isopropanol at 60C° and in 70% Acetone at 4C°. Then, these samples were washed for 5 min. in distilled water at room temperature. All HOPE samples were pre-treated with a 100 mM Tris and 50 mM EDTA solution at 95C° for 5 min and then cooled down for 15C°. The set of samples, selected for enzymatic digestion, was digested by Digest-All III (dilution 1:5) at 37C° for 10 min. The remaining samples were left untreated by Digest-All III. *ERG* FISH probes for all samples were prepared as described above and denatured at 73°C for 5 min, and immediately placed on ice. Posthybridization washing was done with 2 x SSC at 75°C for 8 min., and the fluorescence detection was carried out as previously described. All slides were then counterstained with 4’,6-Diamidin-2’ phenylindoldihydrochlorid (DAPI) and mounted.

FISH protocol for frozen specimen

The mounted specimen were fixed in Acetone for 10mins and were then exposed to Acetone:Methanol (dilution 1:1) for 1 min. These slides were further treated with a 100 mM Tris and 50 mM EDTA solution at 37C° for 30 min. and digested with Digest-All III (dilution 1:2) at 37°C for 5 min. *ERG* FISH probes and clones were prepared as mentioned above. Posthybridisation washing was done with 2x SSC at 75°C for 5 min., and the fluorescence detection was carried out using streptavidin-Alexa-594 conjugates (dilution 1:200) and anti-digoxigenin-FITC (dilution 1:200). Slides were then counterstained with 4’,6-Diamidin-2’ phenylindoldihydrochlorid (DAPI) and mounted.

FISH analysis of FFPE, HOPE, and fresh-frozen tissues

The samples were analysed under an 63x oil immersion objective using a fluorescence microscope (Zeiss, Jena, Germany) equipped with appropriate filters, a charge-coupled device camera and the FISH imaging and capturing software Metafer 4 (Metasystems, Altlussheim, Germany). All cases were independently assessed by two experienced evaluators (M.B., and S.P.) At least 100 nuclei per case were evaluated. Criteria for a efficient FISH staining were clear distinguishable cell morphology with strong distinct *ERG* signals within the nuclei, and minimal background signals.

DNA extraction

DNA extraction was performed using the routine Phenol:Choroloform: Isoamylalcohol (PCI) method. For FFPE and HOPE samples, 5 µm sections were mounted on slides. These were then pretreated with xylene and ethanol wash for deparafinization and were allowed to dry. The samples were then scraped into an eppendorf vial using a sterilized scalpel. Fresh-frozen samples were cut into 5 µm sections and placed directly into the eppendorf vials. Proteinase K (7.5 AU, Qiagen) was then added to all samples and left overnight for protein digestion. The following day, the enzyme was deactivated and the samples were treated with PCI (dilution 25:24:1). The DNA was pelleted using 75% ice cold ethanol. DNA concentration and integrity was estimated using a NanoDrop 1000C Spectrophotometer (NanoDrop Technologies, Wilmington, DE, USA). For subsequent DNA quality assessment using PCR, we chose 6 FFPE specimens, along with their corresponding HOPE and fresh-frozen samples, each having a sufficient amount of DNA and a high integrity value for all the three preservation methods.

PCR

For the assessment of amplifiability and integrity of DNA samples a control gene PCR was performed using five control genes exon and five primer sets for obtaining PCR products ranging from 100 to 600bp.

The following target genes were selected: human thromboxane synthase gene (TBXAS1, exon 9; GenBank accession no. D34621), human recombination activating gene (RAG1, exon 2; GenBank accession no. M29474), human promyelocytic leukemia zinc-finger gene (PLZF, exon 1; GenBank accession no. AF060568), and human AF4 gene (exon 3; GenBank accession no. Z83679, and exon 11; GenBank accession no. Z83687) (van dongen et al). PCR was perfomed using the following primer pairs AF4/X3U GGA-GCA-GCA-TTC-CAT-CCA-GC and AF4/X3L CAT-CCA-TGG-GCC-GGA-CAT-AA (PCR product of 100bp), AF4/X11U CCG-CAG-CAA-GCA-ACG-AAC-C and AF4/X11L GCT-TTC-CTC-TGG-CGG-CTC-C (PCR product of 200bp), PLZF/X1U TGC-GAT-GTG-GTC-ATC-ATG-GTG and PLZF/X1L CGT-GTC-ATT-GTC-GTC-TGA-GGC (PCR product of 300bp), RAG1/X2U TGT-TGA-CTC-GAT-CCA-CCC-CA and RAG1/X2L TGA-GCT-GCA-AGT-TTG-GCT-GAA (PCR product of 400bp), TBXAS1/X9U GCC-CGA-CAT-TCT-GCA-AGT-CC and TBXAS1/X9L  GGT-GTT-GCC-GGG-AAG-GGT-T (PCR product of 600bp).

The AF4/X3L primer was at 2.5 pmol concentration and the rest of the primers were at 1.25 pmol concentrations. We reduced the presence of PCR inhibitors (such as formalin and other fixatives residuals) in HOPE and FFPE isolated DNA by sufficient dilution of all DNA samples. We diluted our DNA samples to an optimal concentration of 100ng/ µl which showed an increased efficiency of amplification.

Cycling conditions were 7 min at 95°C, 35 cycles each with 45 sec at 95°C, 45 sec at 60°C, 1 min and 30 sec at 72°C and a final elongation step of 10 min at 72°C using AmpliTaqGold (Roche, Germany) . This was followed by a gel electrophoresis to analyse the bands of the corresponding PCR products. As reported by Van Dongen *et al*. the presence of five distinct bands for the control genes, ranging from 100-600bp, is a good representation of DNA integrity and amplifiability.

Quantitative RT-PCR

Quantitative RT-PCR analysis was performed with the Roche Light Cycler 480 (Roche, Switzerland). cDNA was synthesized by reverse transcriptase (Revert Aid H Mini First Strand cDNA Synthesis Kit, Fermentas Life Sciences) using 100ng of total RNA and an oligo dT15 primer provided in the kit. We quantified three house-keeping genes (TBP, GAPDH, ß-actin) with varying amplicon lengths to check for RNA integrity from FFPE, HOPE and fresh frozen samples.

Tata box-binding protein (TBP – NCBI accession number NM_003194.4): PCR was performed using the primers 5’-GCC-CGA-AAC-GCC-GAA-TAT-3’, 5’-CCG-TGG-TTC-GTG-GCT-CTC-T-3’ and 5’-FAM-ATC-CCA-AGC-GGT-TTG-CTG-CGG-TAMRA-3’. This results in a 73bp fragment.

Glyceraldehyle-3-phosphate dehydrogenase (GAPDH- NCBI accession number NM_002046.3): PCR was carried out using the primers 5’-GAG-TCA-ACG-GAT-TTG-GTC-GT-3’, 5’ TGG-GAT-TTC-CAT-TGA-TGA-CA-3’ and 5’-FAM-CAA-TGA-CCC-CTT-CAT-TGA-CC-TAMRA-3’. This results in a PCR product of 201bp.

ß-actin (ACTB- NCBI accession number NM_001101.3): The PCR was performed using the primers 5’- AGA-GCT-ACG-AGC-TGC-CTG-AC-3’, 5’-AGT-ACT-TGC-GCT-CAG-GAG-GA-3’ and 5’-FAM-CTG-TAC-GCC-AAC-ACA-GTG-CT-TAMRA-3’. This results in a 300bp fragment.

Using 4 μL diluted cDNA in a 20μL final reaction mixture, Taqman PCR was performed using the following protocol: 10 minutes at 40°C, 10 minutes at 95°C, 50 cycles each consisting of 95°C for 15 seconds and 60°C for 1 minute. Data were analyzed with reference to the cycle threshold (C_t_). This value denotes the cycle at which the fluorescence from a sample crosses the background threshold. All reactions were performed at least twice in duplicates.

Protein extraction:

For the fixed (FFPE, HOPE) samples sections ranging from 3-8 x 10 µm were used for protein extraction, depending on the size of the area of interest. For the fresh frozen tissues 10-15 x 20 µm sections were used which were directly transferred into a reaction tube. As described previously, after deparaffinization of the sections, the areas of interest were scratched (according to an HE stained reference section) from adjacent unstained slides with a needle and transferred into EXB Plus buffer (Qiagen, Hilden, Germany). The buffer volumes varied from 50 µl up to 100 µl, depending on the size of the procured tissue areas. Proteins were extracted according to the protocol provided by the manufacturer and stored at -20°C.

For the fresh frozen tissues 10-15 x 20 µm sections were used which were directly transferred into a reaction tube. We gave the EXB Plus extraction buffer on top of the frozen sections and crushed the material by pestling and shaking. Then we continued according to the protocol for fixed tissues, i.e heating for 20 min at 100°C and incubation for 2 hours at 80°C. After centrifugation the lysates were stored at -20°C.

Protein concentrations were determined using the Bradford protein assay (BioRad Protein Assay (# 500-0006)).

Western blot:

Proteins were separated by 10% SDS-polyacrylamide gel electrophoresis followed by transfer to nitrocellulose filters (Schleicher and Schuell, Dassel, Germany). From each sample 18 µg of protein lysate were loaded. We blocked the membrane with 5% milkpowder/ TBST for one hour at room temperature and incubated the antibody Golph2 (clone 5B10, Abnova # H00051280-M06) in a dilution of 1:1000 in 5% milkpowder /TBST at 4°C over night. The secondary antibody (anti-mouse) was used in a dilution of 1:10.000 in 5% milk powder/ TBST for one hour at room temperature. Immunoblots were visualized with ECLplus (Amersham/ GE Healthcare). The blots were washed with TBST and incubated again with ß-Actin antibody (Sigma A1978 AC15) in a dilution of 1:10.000 in 5% milkpowder/TBST as a loading control and once more with a PSA antibody (Novocastra # NCL-PSA-431) used 1:1000 in 5% milkpowder/TBST. The same secondary anti-mouse antibody as mentioned above was used.
